# Celebrating International Women’s Day: where does this leave the paramedic profession?

**DOI:** 10.29045/14784726.2022.03.6.4.1

**Published:** 2022-03-01

**Authors:** Caitlin Wilson, Larissa Stella Prothero, Julia Williams

**Affiliations:** University of Leeds; North West Ambulance Service NHS Trust ORCID iD: https://orcid.org/0000-0002-9854-4289; East of England Ambulance Service NHS Trust ORCID iD: https://orcid.org/0000-0002-5440-8429; South East Coast Ambulance Service NHS Foundation Trust; University of Hertfordshire ORCID iD: https://orcid.org/0000-0003-0796-5465

International Women’s Day 2022 has adopted the theme #BreakTheBias. It is encouraging people to look at how we can break the bias in our communities, in the education system and in the workplace. It promotes a vision of a gender equal world – one where diversity is celebrated and differences are valued. With growing numbers of women working in unscheduled, urgent and emergency care settings, what progress are we making within our working roles?

Of the 1.3 million staff employed by the NHS, more than 75% are women, but how many work in UK ambulance settings? The ambulance workforce has been traditionally dominated by men; however, times are changing and now women represent 42.5% of ambulance staff across all service roles (NHS England, 2021). For UK paramedics, the Health and Care Professions Council (HCPC) reports 41.7% of paramedic registrants to be female and our profession remains the only one with more male than female registrants (HCPC, 2021). Looking to the future, hopefully this gender imbalance will be addressed by the increasing numbers of women on pre-registration degree programmes across the UK.

But do women think of the health challenges they may face when entering the paramedic profession? There is evidence which shows shift-work negatively impacting both mental and physical health, including female reproductive health – that is, menstruation, pregnancy and the menopause (Harrington, 2001). Night shifts and long working hours can alter a woman’s circadian rhythm, affect hormone levels and disrupt the menstrual cycle. For women of child-bearing age, shift-working has been linked to increased risk of spontaneous abortion, low birth weight and prematurity (Fernandez et al., 2016; Stock et al., 2019). All women will experience a menopause transition when their oestrogen levels decline, and their menstrual periods cease. This typically occurs at 51 years; however, up to 10% of women can experience early menopause or premature ovarian insufficiency, which are both associated with typical menopausal symptoms (National Health Service, 2018). Also, transgender, non-binary and intersex people can experience the menopause. We should not forget our male colleagues in this discussion. As they work alongside us as crewmates or office colleagues, they too will experience our health challenges, and some will experience the male menopause as their testosterone levels fall (National Health Service, 2019). Menopausal symptoms can be challenging, and impact on personal well-being, workplace attendance and performance. Employer consideration of flexible working; maternity, paternity and adoption leave; childcare arrangements; alternative roles; and improved staff support may allow women and men to successfully balance work and family life and remain valued members of the ambulance workforce before retirement. Currently, there appears to be a paucity of evidence as to why and at what age women (and men) leave the ambulance profession and this is an area that would benefit from further exploration.

Linked to this is a need for more research on the daily experiences of women working in the ambulance setting. Bullying and harassment, including sexual harassment, have been reported in ambulance services in the UK and internationally. The ambulance profession has been referred to as a ‘boys’ club’ culture that is resistant to change (Manolchev & Lewis, 2021). In some countries there are examples of women-only ambulance services that provide female healthcare to communities with specific cultural requirements (*Arab News*, 2017; Julian, 2014). Understanding the roles, responsibilities and experiences of ambulance women in diverse clinical settings will enable appropriate support resources to be developed and female working lives to be improved.

When we begin to look at leadership roles within ambulance services and across the broader NHS, these positions are predominantly held by men (NHS Digital, 2018). Figures from the UK Government (2021) Gender Pay Gap Service suggest that across ambulance services in England, women occupy lower paid jobs compared to men. This is illustrated by women making up on average only 42.8% of the highest hourly pay quarter, while the other quarters are split 50:50. This gap is narrowing within ambulance services and across the NHS over time, but more change is needed to support women to take up these leadership positions – ideally supported by research.

Speaking of research, this is one avenue of career progression for paramedics and one that is increasingly gaining traction in the UK. While no data are collected on the gender of research paramedics or ambulance staff pursuing clinical academic careers, the gender split of research leads in UK ambulance services is similar to that of senior positions overall: five out of 13 research leads are women. However, it is inspiring that the College of Paramedics head of research is a woman, and here on the *British Paramedic Journal* editorial board, both women and men (3:5) are represented.

But what about when it comes to disseminating our research? Nowadays, conference organisers consider a balanced selection of speakers – be that of gender, ethnicity, topic areas or roles. And what about publications? The *BPJ* editors have recognised this journal does not collect author demographic information (including gender), so cannot report this information. We are now discussing the introduction of a voluntary gender-identity question for authors. This will enable us to report author gender and relevant gender-related trends in our research publications.

Lastly, while the focus of this International Women’s Day editorial is on women in the ambulance service and paramedic research, it is not our intention to dismiss the challenges of men and non-binary individuals in the ambulance and research workforce. We recognise the need to work together to advance the evidence-base for the whole paramedic profession.

## Author contributions

CW and LSP are joint first authors as they developed the initial draft for this manuscript. All three authors jointly revised the manuscript for publication. All three authors are on the *BPJ* editorial board.
